# Identification of Neurophysiological Markers of Verbal Information Processing Using Cognitive Evoked Potentials for Studying Schizophrenia Spectrum Disorders

**DOI:** 10.17691/stm2022.14.6.06

**Published:** 2022-11-28

**Authors:** N.S. Nuzhina, P.A. Prodius, I.V. Mukhina

**Affiliations:** Assistant, Department of Normal Physiology named after N.Yu. Belenkov; Privolzhsky Research Medical University, 10/1 Minin and Pozharsky Square, Nizhny Novgorod, 603005, Russia;; Associate Professor, Department of Normal Physiology named after N.Yu. Belenkov; Privolzhsky Research Medical University, 10/1 Minin and Pozharsky Square, Nizhny Novgorod, 603005, Russia; Associate Professor, Department of Physiology and Anatomy, Institute of Biology and Biomedicine; Lobachevsky State University of Nizhni Novgorod, 23 Prospekt Gagarina, Nizhny Novgorod, 603950, Russia; Professor, Head of the Department of Normal Physiology named after N.Yu. Belenkov; Privolzhsky Research Medical University, 10/1 Minin and Pozharsky Square, Nizhny Novgorod, 603005, Russia; Professor, Department of Neurotechnologies, Institute of Biology and Biomedicine; Lobachevsky State University of Nizhni Novgorod, 23 Prospekt Gagarina, Nizhny Novgorod, 603950, Russia

**Keywords:** schizotypy, cognitive evoked potentials, processing of visual verbal information, semantic categorization, control of mental activity

## Abstract

**The aim of the study** is to identify neurophysiological markers of information processing sensitive to verbal thinking impairment in persons with schizotypal personality organization using the method of cognitive evoked potentials.

**Materials and Methods:**

Cognitive evoked potentials were studied in 40 students of higher school with high and low (control) scores obtained on the Schizotypal Personality Questionnaire (SPQ) in response to the presentation of visual verbal information in the form of agreed word-combinations. In the first series of presentations, it was necessary to refer the attribute to one of the categories and to read the noun silently (non-target condition). In the second series, the attribute should be read silently, and then the following noun has to be categorized (target condition). There has been performed a cluster analysis of the evoked potential curves obtained in response to the noun presentation in the target and non-target conditions in the groups of participants with high and low scores gained on SPQ.

**Results:**

Processing of the verbal stimulus under passive reading conditions and under the conditions of word categorization within the frameworks of a simple context has shown that in the group with low scores on the schizotypal questionnaire, lateralization of the N150 component to the left side was observed in contrast to the group with high scores. In this group, increase of the N400 component amplitude was found in response to the words presented for their passive reading in comparison with the categorization condition. On the contrary, in the group with high schizotypy scores, the N400 component appeared to be insensitive to the type of the task, i.e. neurophysiological differences were not expressed between reading and performing stimuli categorization task. These persons were found to have the decreased amplitude of the late positive component as compared to the control group under the condition of passive reading of the words. Increase of the late negative wave amplitude was registered in response to the target words subjected to categorization in comparison with reading in both groups of participants. Insufficiency of language lateralization and deficit connected with the language networks being activated automatically at the initial stage of word recognition are observed in the examined persons with schizotypy. Additionally, processing of the information in these persons at the stage of lexical-semantic processes is weakly modulated by the task imposing different requirements on the level of mental activity control. Some reduction of mnestic process activation is also possible, which is manifested during passive reading of the words, but not in the conditions of their categorization.

**Conclusion:**

The research conducted may serve as a starting point for a more detailed and long-term study of the fundamental mechanisms of impairment in information processing in the course of clinically evident pathology formation. In the applied aspect, the work may be considered as a contribution to the ongoing search for neurophysiological markers for early diagnosis of schizophrenia spectrum disorders.

## Introduction

The concept of “schizotypy” characterizes a specific personality organization with manifestations similar to those which are observed in schizophrenia spectrum disorders but is less intensive [1]. People with high scores in psychometric schizotypy indices have been shown to have an increased risk of developing schizophrenia and psychoses associated with schizophrenia [2]. In this regard, the concept of schizotypal personality organization is interesting as an initial link of the base for investigating the process of schizophrenia spectrum disorders formation, deeper understanding of specific aspects of mental activity and cognitive processes going on in this continuum of disorders.

Thought disorder is known to be a characteristic feature of schizophrenia [3] and schizotypy [4]. The cognitive operations may be reflected by registration of brain evoked potentials representing evoked electrical neuron activity related to the stimulus processing. Traditionally, N400 and late positive components of evoked potentials are considered for the assessment of verbal information processing.

The N400 component is a negative wave forming a peak around 400 ms post-stimulus onset. This wave is associated with context-dependent processes of lexical retrieval [5] or difficulties of semantic integration of the words into the context [6]. The component is also sensitive to the relevance of the stimuli to the task and is more pronounced in amplitude in response to the task-incongruent stimuli [7]. The literature data speak of a high sensitivity of the N400 component to thinking activity disorder both in schizophrenia and schizotypy. The deficit of semantic stimuli processing in people with schizophrenia and schizotypal personality organization manifests as the reduction of the semantic priming effect [8-13], although there are studies reporting the increase of the priming effect in incongruent stimuli in case of more automated information processing [14-16].

The late positive component (500–700 ms post-stimulus onset) is associated with functioning of the episodic memory, memorizing previously presented information, formation of memory traces, the component amplitude at that increases in case of repeated presentation of the stimuli and successful recognition of the memorized stimuli by the examined person [17-20]. The vast majority of studies demonstrate the reduction of the late positive component amplitude in schizophrenia spectrum disorders [8, 13, 21].

Late negative components remain less studied, nevertheless it is known that changes in the electrical activity are observed at the later temporary stages while processing verbal material (700–1500 ms) in response to presentation of the target words relative to the non-target stimuli [22, 23]. The N700 component may be distinguished among the late negative components which may show sensitivity to the target and non-target presentation conditions. It is associated with a supplementary functioning of attention and memory after conscious stimulus recognition. The component amplitude increases when the stimulus attracts attention or when it is memorized and, conversely, it decreases in passive perception [24, 25].

In our investigation, the task was set to study the characteristics of evoked potential components at various stages of processing verbal information by the brain: in more automated conditions and in the conditions requiring activation of cognitive control mechanisms. We were also to detect possible changes of these characteristics in persons with schizotypal personality organization since this aspect remains insufficiently studied and data presented in the literature are contradictory. Thus, some researchers confirm the phenomenon of hyperactivation in the semantic networks in the condition of a more automated processing of information in persons with schizotypal personality disorder [26]. Others reveal alterations in voluntary regulation and do not find them during a more automated processing in people with high schizotypy [27]. Kiang et al. [12] demonstrate the reduction of priming effect in persons with a high level of schizotypy in the conditions of a more automated, as well as more voluntary processing of information.

Passive reading of verbal information, not implying fulfillment of additional tasks, seems to be a more automated process using primarily mechanisms of descending control [28]. The task of semantic categorization of the presented stimuli, in its turn, activates voluntary control. A similar factor of task variation was used in the linguistic paradigm during auditory speech perception [29]. We used this factor in our work in respect to processing of visual verbal information and investigated cognitive evoked potentials (CEPs) in the conditions of more automated processing of verbal stimuli (passive reading) and in the conditions requiring activation of the systems of cognitive function control (semantic categorization). Words in minimal compositional context were used as verbal stimuli, i.e. word combinations, since their processing allows one to detect changes which are not observed when isolated words or words which are not connected compositionally with each other, are processed [30].

Our investigation was not directed to the analysis of average amplitude values of specific evoked potential components. Using cluster analysis, we attempted to identify those stages of information processing which may seem to be sensitive to the change of the task within the group and also demonstrate intergroup differences in the target and non-target presentation of a verbal stimulus. Additional knowledge about the conditions in which this or that component of evoked potentials show sensitivity to the specific character of verbal stimulus processing in people with schizotypal personality organization is believed to be the necessary contribution to the search for neurophysiological markers of schizophrenia spectrum pathology formation.

**The aim of the study** is to identify neurophysiological markers of information processing sensitive to verbal thinking impairment in persons with schizotypal personality organization using the method of cognitive evoked potentials.

## Materials and Methods

Students of the natural-scientific profile aged from 19 to 25 years with normal and corrected vision took part in the study. To conduct the study, permission from the local Ethics Committee of Privolzhsky Research Medical University (Nizhny Novgorod, Russia) was obtained. The students were pre-informed about the study procedure and agreed to participate in it.

In order to reveal persons with schizotypal organization, the Schizotypal Personality Questionnaire (SPQ) [31] was used. The results of screening of 185 students have been analyzed. By the number of points scored, the median equal to 20 points was derived. According to this value, participants with the score of 19.5 points and less were included into the control group (n=20). It was decided to refer the participants who scored more than 30 points to the group with high scores (n=20) in order to analyze the data obtained from the persons with more evident signs.

Evoked potentials were registered using Neuron-Spectrum-4/EPM multifunctional computer complex (Neurosoft, Russia) at the frontal (Fр1, Fр2, F7, F8, F3, F4, Fz), central (C3, C4, Cz), parietal (P3, P4, Pz), and posterior temporal (Т5, Т6) sites of active electrodes. Two ipsilateral referents were employed: one of them was located on the right and the other on the left mastoid process of the temporal bone. A ground electrode was placed on the vertex. Electrode impedance was below 10 kOhm.

The participants were offered two series of verbal stimuli, each of them included presentation of 60 pairs of words representing agreed phrases. An average length of the presented nouns in the first series was 6.03±0.97 letters, in the second — 5.72±1.14. A mean frequency of noun use in the first series was equal to 19.64±18.16 per million, in the second series it was 16.78±23.16 per million [32]. In the first series of presentations, it was necessary to refer the attribute to one of the categories (expressing qualitative or ordinal characteristic) and to read the noun silently. In the second series, the attribute has to be read silently and to refer the noun to one of the semantic categories (instruments or interior). Each word was presented on the screen during 1200 ms. When a question mark appeared, an appropriate key should be pressed on a keyboard to give an answer. Nouns were non-target stimuli in the first series, and target stimuli in the second one.

Oculographic, myographic, motor, and other artifacts were manually removed from the original EEG recordings. Intervals from 0 to 1200 ms were used for averaging after stimulus presentation. A number of averages per every type of stimulus for each examined person ranged from 30 to 40. In the groups of participants with high and low SPQ scores, evoked potential curves in response to the presentation of the nouns in the conditions of categorization (target condition) and passive reading (non-target condition) were compared.

### Statistical analysis

Data were statistically processed using SPSS Statistics 22.0 program and MNE 23.0 package for the Python 3.9. programming language. Normality of distribution was checked with the help of Kolmogorov–Smirnov test. In order to detect spatial-temporal patterns of evoked potential differences, a non-parametric cluster analysis was applied [33], implemented in the MNE 23.0 package for Python 3.9 language. Correction for multiple comparisons was performed by means of the permutation test [33]. A threshold of p=0.05 was employed for cluster formation. The level of cluster statistical significance was determined based on 10,000 permutations. Differences were considered statistically significant at p<0.05. The results were represented as Ме [Q1; Q3].

## Results

### Behavioral data

An average response time to the target nouns in the examined persons with high SPQ scores was 0.45 [0.36; 0.66] ms, while in the participants with low scores it was 0.48 [0.33; 0.65] ms; no statistically significant differences between the groups were found (p>0.05).

### Electrophysiological data

Statistically significant amplitude differences between Т5 and Т6 electrodes (p<0.05) for the negative component in the interval from 157 to 220 ms were obtained in the group with low SPQ scores in the non-target condition. Lateralization of the component to the left side was observed. In the group with high scores, no interhemispheric differences were detected.

Comparing the components of evoked potentials in the group with low SPQ scores, statistically significant differences were established between the CEP curves in the conditions of categorization and passive reading for the N400-related clusters (Table 1), at the central (C3, C4, Cz, p<0.01), parietal (P3, P4, Pz, p<0.01), and right posterior temporal (Т6, p<0.01) electrodes. The component amplitude was higher in response to the non-target stimuli than to the target ones. In the target and non-target conditions in this group, statistically significant interhemispheric amplitude differences were obtained at the Т5 and Т6 electrodes (p<0.05) in the N400-related clusters. At the same time, lateralization of the component to the right side was observed. Comparison of the CEP curves in the group with high SPQ scores did not show statistically significant differences between the amplitude of the N400 wave in the target and non-target conditions of presentation, as well as between the amplitude of this wave in the right and left hemispheres.

**Table 1:** The results of cluster analysis obtained by comparing the curves of cognitive evoked potentials (CEPs) in the target and non-target conditions in participants with low and high SPQ scores

Electrodes	Low-score group				High-score group			
	CEP interval (ms)*	Ме [Q1; Q3]		p (after correction for multiple comparisons)	CEP interval (ms)*	Ме [Q1; Q3]		p (after correction for multiple comparisons)
		Target word	Non-target word			Target word	Non-target word	
F3	679–814	0.44 [0.16; 0.63]	1.05 [1.0; 1.50]	0.0078				
C3	235–380	–1.61 [–1.69; 0.32]	–2.80 [–3.21; –1.86]	0.001	767–874	–0.61 [–0.74; –0.42]		0.0056
	660–887	–0.44 [–0.59; –0.01]	0.68 [0.40; 0.91]	0.0002		0.39 [0.36; 0.48]		
C4	241–379	–1.39 [–1.52; –0.28]	–2.51 [–2.73; –1.98]	0.0044	766–887	–0.38 [–0.60; –0.16]	0.63 [0.61; 0.67]	0.0056
Cz	199–369	–1.56 [–2.47; 1.03]	–3.27 [–3.81; –1.01]	0.0004				
	663–824	–0.01 [–0.27; 0.48]	1.29 [1.19; 1.73]	0.0006				
P3	236–378	–0.004 [–0.68; 1.86]	–2.11 [–2.49; –0.39]	0.0018	717–951	–0.72 [–0.85; –0.56]		0.0002
	657–909	–1.0 [–1.19; –0.64]	0.47 [0.31; 0.65]	0.0002		0.37 [0.22; 0.74]		
P4	238–382	–0.17 [–0.92; 1.18]	–1.87 [–2.52; –1.08]	0.001	737–925	–0.37 [–0.60; –0.13]		0.0002
	701–939	–0.82 [–0.95; –0.67]	0.35 [0.24; 0.43]	0.001		0.56 [0.46; 0.65]		
Pz	235–383	–0.50 [–0.60; 1.06]	–2.41 [–2.70; –1.94]	0.0008	797–940	–0.84 [–1.04; –0.76]		0.002
	741–902	–1.25 [–1.33; –1.17]	0.44 [0.29; 0.47]	0.0024		0.45 [0.29; 0.61]		
T5	813–907	–0.97 [–1.12; –0.80]	0.14 [–0.01; 0.32]	0.0178	848–964	–043 [–0.48; –0.37]	0.23 [0.21; 0.30]	0.0054
T6	267–393	0.40 [–0.08; 1.26]	–0.75 [–0.96; 0.08]	0.0056	823–924	–0.35 [–0.38; –0.29]		0.024
	798–993	–0.52 [–0.58; –0.35]	0.11 [0.08; 0.18]	0.0012		0.30 [0.29; 0.38]		

* Presented are only those intervals in which statistically significant differences were identified in cognitive evoked potential curves (p<0.05) in response to presentation of target and non-target nouns. Lines containing no data designate that statistically significant differences at these electrodes were not detected.

**Figure 1.**
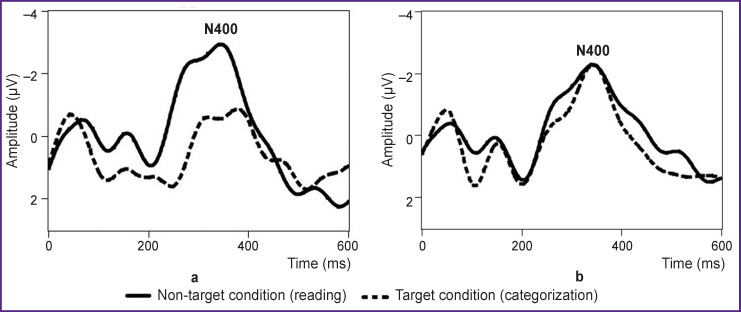
Averaged curves of evoked potentials observed in the groups with low and high SPQ scores in response to presentation of nouns in two experimental conditions: (a) averaged curves of evoked potentials (n=20) at the middle parietal electrode (Pz) in the low-score group according to SPQ; (b) averaged curves of evoked potentials (n=20) at the middle parietal electrode (Pz) in the high-score group according to SPQ

The evoked potential curves for the target and non-target conditions in the groups with high and low SPQ scores are exemplified in Figure 1.

In the non-target condition, statistically significant amplitude differences (p<0.05) in these groups were established in the cluster, to which the component N400 corresponds (F3 electrode, 290–378 ms), the wave amplitude in this case turned out to be more pronounced in the low-score group as compared to the group with high SPQ scores (Table 2).

**Table 2:** The results of cluster analysis obtained by comparing the curves of cognitive evoked potentials (CEPs) in participants with low and high SPQ scores in the target and non-target conditions

Electrodes	Non-target condition				Target condition			
	CEP interval (ms)*	Ме [Q1; Q3]		p (after correction for multiple comparisons)	CEP interval (ms)*	Ме [Q1; Q3]		p (after correction for multiple comparisons)
		Low SPQ scores	High SPQ scores			Low SPQ scores	High SPQ scores	
F3	290–378	–3.52 [–3.66; –3.23]	–2.31 [–2.47; –2.07]	0.0378				
	517–614	1.91 [1.51; 2.01]	0.38 [0.29; 0.72]	0.0289				
F4	523–606	1.93 [1.69; 1.98]	0.45 [0.42; 0.60]	0.0374				
C3	455–603	1.64 [1.56; 2.09]	0.42 [0.26; 0.84]	0.0038				
C4	458–593	1.56 [1.29; 1.78]	0.22 [0.14; 0.50]	0.0054				
Cz	456–603	2.07 [1.70; 2.81]	0.82 [0.48; 1.03]	0.006				
P4					675–779	–0.52 [–0.93; –0.21]	0.28 [0.01; 0.68]	0.294

* Presented are only those intervals in which statistically significant differences (p<0.05) were identified in the groups of participants with low and high SPQ scores. Lines containing no data designate that statistically significant differences at these electrodes were not detected.

**Figure 2.**
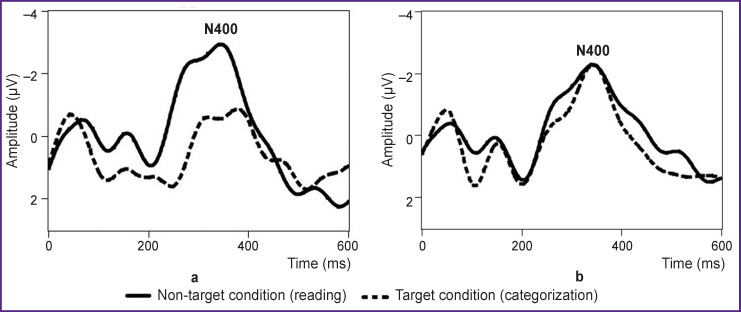
Averaged curves of evoked potentials (n=20) at the right central electrode (С4) observed in the groups with low and high SPQ scores in response to presentation of nouns in the non-target condition

In the process of passive reading, evoked potential curves differed statistically significantly in the selected groups in the clusters, to which the late positive component corresponds at the frontal (F3, F4, p<0.05) and central (C3, C4, Cz, p<0.01) electrodes (see Table 2). In this case, the greater component amplitude was observed in the low-score group as compared to the high-score group. Intergroup differences for the late positive component in the non-target condition are shown in Figure 2. In the target condition of presentation, statistically significant intergroup differences for the component amplitude were not identified.

In the low-score group, statistically significant differences were detected between the CEP curves in the conditions of categorization and passive reading for the clusters related to the late negative wave (see Table 1) at the left frontal (F3, p<0.01), central (C3, Cz, p<0.01), parietal (P3, P4, Pz, p<0.01), and posterior temporal (Т5, p<0.05, Т6, p<0.01) electrodes. In the group with high SPQ scores, statistically significant differences between the CEP curves were also detected in the given conditions for the clusters related to the late negative wave at the central (C3, C4, p<0.01), parietal (P3, P4, Pz, p<0.01), and posterior temporal (Т5, p<0.01; Т6, p<0.05) electrodes. The component amplitude was greater in the conditions of stimulus categorization as distinct from passive reading in both examined groups. In the target condition, intergroup difference was found at the P4 electrode (p<0.05) in the cluster corresponding to this component (see Table 2); at the same time, the amplitude of the late negative wave appeared to be more pronounced in the group with low SPQ scores as compared to the high-score group.

## Discussion

No statistically significant intergroup differences for early components have been identified in our work, which is in agreement with other investigations of schizophrenia spectrum disorders in linguistic paradigms [26]. But it should be noted that the works in other non-linguistic paradigms report about changes of exogenous components P50, N100, N200, Р200 in patients with schizophrenia [34-37]. The analysis of interhemispheric asymmetry has shown that in the low-score group, the amplitude of the negative component with a peak latency of about 150 ms turned out to be more pronounced at the left posterior temporal electrode in comparison with the right side during passive reading. This is in line with the literature data, i.e. at the electrophysiological level, the first evoked component reflecting automatic word recognition is a left-sided N150 [38]. Interhemispheric differences for the given component were absent in the group with high schizotypy scores. This agreed with the data about insufficient left-sided lateralization for the N150 component in patients with schizophrenia, which is explained by the absence of hemispheric specialization of linguistic networks which are automatically activated at the earliest phase of word recognition [38]. Our results may also indicate to the insufficiency of language lateralization in persons with schizotypal personality traits and to the deficit related to those linguistic networks which are activated at the initial stage of word recognition. Thus, absence of interhemispheric asymmetry may become a sensitive marker for identifying changes in verbal information processing in persons with schizotypal personality organization.

In the group with low schizotypy scores, amplitude increase was identified in the N400 component in response to the words in the conditions of their passive reading compared to the categorization condition. Increase of the N400 amplitude in response to the non-target stimuli agrees with the study performed by Mar’ina and Strelets [7] demonstrating increase of the component amplitude in response to the task-irrelevant stimuli. The effect of incongruence to the task was likely to be observed in our study also in relation to the words in the non-target condition (reading) when they were perceived as task-irrelevant and demonstrated a higher amplitude in comparison with the target words. However, it has been established by us that in the high-score group there were no statistically significant differences in the N400 amplitude between categorization and reading of the nouns, i.e. the effect of N400 was absent. From the point of view of neurophysiology, it designated that in the group of participants with schizotypal personality traits, the resources spent on processing of the word, which has to be simply read, appeared to be analogous to the effort to process the stimulus subjected to categorization. Most probably, information processing in schizotypy is weakly modulated by the difference in the level of control of mental activity. It may well be that sensory information from the environment in persons with schizotypal personality organization is processed less passively and combines quickly with the notions, other sensory impressions, and anticipation stored in the memory, i.e. activation of descending regulation mechanisms has a wider influence on the perception of passively read information. Intensification of descending processing and its impact on the more automated processes are considered in patients with schizophrenia [39-41]. The deficit of inhibiting redundant information and, as a consequence, reduction of the N400 effect in people with a high schizotypy level are observed also in the investigations dealing with lexical-semantic features [11-13].

However, it is interesting to compare our results with other research performed by Mar’ina et al. [42], in which preservation of task-incongruency effect was observed in patients with schizophrenia. These data do not agree with the results of our work probably due to the differences in the study design. Our model used a simple context in the form of word combinations rather than separate words as in the abovementioned study. A word in the context elicits more associations and activates more connections in the semantic memory, which is very likely to result in the excessive processing of the stimuli in the process of their passive perception in people with schizophrenia spectrum disorder. This is consistent with the fact that excessive associations not connected with the task and a violation in the use of a context are characteristic for schizotypy [11, 43].

Besides, the N400 amplitude appeared to be more pronounced at the right posterior temporal electrode during passive reading of the words in comparison with the left side in low-scored participants. Some literature sources report that this component may have a right-sided lateralization [44, 45]. In our study, interhemispheric differences for the N400 component were absent in the group with high schizotypy scores. However, it is unlikely that this change in lateralization of N400 may be a biomarker, since generators of this component are traditionally found in the left hemisphere [46].

The amplitude reduction of the late positive component was observed in the participants with schizotypal personality traits in the condition of reading the words, which is consistent with the results of previous investigations both in clinically evident schizophrenia [8, 21] and in the group with a high level of schizotypy among healthy people [13]. It may be supposed that identified amplitude differences designate the deficit of episodic memory in the examined participants with high schizotypy scores. The deficit of episodic memory is known to be in patients with schizophrenia [47], in healthy relatives of such patients [48], and in people with schizotypy [49]. However, the results obtained for this component should be interpreted from the point of view of mnestic processes with great care, as our paradigm was not targeted at studying the processes related to memory. There are data showing that P600 component may be modulated by the level of attention to the stimulus [50, 51]. The amplitude reduction of this component in persons with schizotypal personality traits is likely to reflect the decrease of the current level of sustained attention to the non-target stimulus relative to the control group. It should be noted that in the second series of presentations, when the task was to categorize the words, the component did not differ between the groups. Thus, intergroup differences for the late positive wave were identified only in the condition of implicit task, implying passive word reading. In this case, the processes reflected in the late positive component did not demonstrate neurophysiological differences in the participants with schizotypy traits from the control group in the conditions requiring activation of cognitive activity control. Within the groups, the component appeared to be insensitive to the differences in the conditions of stimuli presentation and did not demonstrate differences between the responses to the target and non-target words in both of the groups with different SPQ scores.

A pronounced late negative wave beginning its formation at about 700 ms was registered at the central, parietal, and posterior temporal electrodes for the nouns in the target condition. Moreover, formation of this wave in the target condition was characteristic for both groups irrespective of the scores, i.e. the component was sensitive to the change of the task in the group with schizotypy, as well as in the control group. At the site of the right parietal region, reduction of the amplitude of the late negative wave was noted in response to the presentation of the target words in the high-score group relative to the participants with lower scores. Supplementary functioning of the cognitive control mechanisms connected with the purposeful retention of information in the memory for subsequent response, mobilization of attention, and preparation to the motor response is likely to experience some alterations in persons with schizotypal personality organization.

## Conclusion

The amplitude differences in cognitive evoked potentials obtained in this investigation for the groups with high and low SPQ scores for identification of schizotypal personality traits allow us to select neurophysiological correlates of information processing undergoing changes in persons with schizotypal organization. The obtained data indicate to the insufficiency of language lateralization in the participants with schizotypy and to the deficit associated with those language networks which are automatically activated at the initial stage of word recognition. Besides, information processing at the stage of lexical-semantic processes is weakly modulated by the task presenting different requirements to the level of cognitive activity control. In schizotypy, it is also possible some decrease of mnestic process activation which occurs in the process of passive reading of the words but not in the condition of their categorization. Cognitive control mechanisms connected with the purposeful retention of information in the memory for subsequent response, mobilization of attention, and preparation to the motor response are not practically affected.

Exploration of the stages of visual verbal stimulus processing in a simple context for automated and more controlled conditions seems to be rather promising for disclosing fundamental mechanisms of information processing impairment in the process of formation of clinically evident psychic pathology. In the applied aspect, the perspective of identifying specific neurophysiological markers for early diagnosis of schizophrenia spectrum disorders seems to be interesting.
